# Altered neural processes underlying executive function in occupational burnout—Basis for a novel EEG biomarker

**DOI:** 10.3389/fnhum.2023.1194714

**Published:** 2023-10-02

**Authors:** Mia Pihlaja, Jari Peräkylä, Emma-Helka Erkkilä, Emilia Tapio, Maiju Vertanen, Kaisa M. Hartikainen

**Affiliations:** ^1^Behavioral Neurology Research Unit, Tampere University Hospital, Tampere, Finland; ^2^Faculty of Medicine and Health Technology, Tampere University, Tampere, Finland

**Keywords:** brain, burnout, ERP, EEG, executive function, cognitive control, brain physiology, brain health

## Abstract

**Introduction:**

As burnout has become a global pandemic, there is a call for improved understanding and detection of alterations in brain functions related to it. We have previously reported challenges in executive functions (EFs) in daily life, especially in metacognition, in subjects with occupational burnout, along with alterations in cardiac physiology. In the current study, we focused on the impact of burnout on brain physiology during a task requiring EF.

**Methods:**

Fifty-four volunteers filled in inventories of burnout, depression, and EF in daily life (BBI-15, BDI, and BRIEF-A). Based on the BBI-15 score, subjects were divided into burnout and non-burnout groups. Subjects performed a Go/NoGo test (Executive RT test) engaging several EFs, while their EEG was recorded. The inventory scores, cognitive performance scores, and event-related potential (N2, P3) amplitudes, latencies, and interpeak latencies (IPLs) were compared between the groups.

**Results:**

There were significant differences in the BDI and BRIEF-A scores between the groups, with more symptoms of depression and challenges in daily life in the burnout group. There were no differences in objective performance measures in the EF task between the groups. However, centroparietal P3 amplitude was larger, and while there were no differences in N2 or P3 latencies, N2-P3 IPL was longer in the Go condition in the burnout than in non-burnout group. Both ERP measures correlated significantly with burnout symptoms. A regression model from centroparietal P3 amplitude and N2-P3 IPL predicted significantly both the BBI-15 score and the BRIEF-A metacognition index.

**Discussion:**

We conclude that burnout is linked with challenges in EF in daily life and alterations in the underlying neural processes. While cognitive performance in the task was equal, electrophysiological measures differed between the groups. Prolonged N2-P3 IPL points toward slowed transition from one cognitive process to another. Increased P3 amplitude, on the other hand, reflects increased allocation of neural processing resources. This may be a compensatory mechanism, allowing for equal performance with controls. These electrophysiological measures, obtained during the EF task, show promise as brain physiology-based biomarkers of burnout, contributing to its improved and objective detection. In addition, these results indicate occupational burnout is linked with objective alterations in brain physiology.

## 1. Introduction

Executive functions (EFs) are higher-order control functions that regulate cognition, emotion, and behavior according to current goals and social context rather than habits and impulses (Diamond, 2013). Thus, it is not surprising that most occupations in modern work life rely on efficient executive functions (EFs). On the other hand, ever-increasing cognitive and affective demands at work may contribute to burnout, which has become a global pandemic. The current diagnosis of burnout is mainly based on psychological concepts and lacks an objective assessment of brain physiology and cognitive functions. To that end, there is a huge need for a better understanding of the impact of burnout on cognitive brain functions and a call for improved diagnostics and treatment of burnout.

Burnout remains to be recognized as a neuropsychiatric condition similar to depression. Burnout and depression share partly overlapping symptomatology with both linked to challenges in EF (Peräkylä et al., 2021; Pihlaja et al., 2022). In our recent study, challenges in EF in subjects with burnout were detected using an inventory of EF in daily life (Behavior Rating Inventory of Executive Functions-Adult Version; BRIEF-A; Pihlaja et al., 2022). In our efforts to detangle depression and burnout and their distinct contributions to challenges in EF, we found out that while depression was especially linked with challenges in behavioral regulation (Peräkylä et al., 2021), burnout was linked with challenges in metacognition (Pihlaja et al., 2022). The challenges in metacognition associated with burnout were also linked with alterations in cardiac physiology, specifically decreased heart rate variability. While the long-term alterations in brain physiology and the associated deficits in EF in burnout remain to be established, in a recent study by Wiehler et al. cognitive strain and consequent cognitive fatigue due to a work-day-long engagement in a task challenging EF were linked with higher glutamate levels in the prefrontal cortex and suboptimal decision-making (Wiehler et al., 2022). The observed decline in decision-making was likely linked to a temporarily compromised EF.

Electroencephalography (EEG) and event-related potentials (ERPs) provide useful tools for assessing alterations in brain physiology and neural processes underlying rapid mental events (Polich, 2007; Folstein and Van Petten, 2008). As ERPs allow for objective detection of brain dysfunction, they may provide a basis for novel biomarkers of burnout. There is a call for such objective brain physiology-based biomarkers of burnout due to a common discrepancy between the magnitude of subjectively reported symptoms and minimal or lacking objective findings of impairment. Subjects with burnout frequently report significant subjective cognitive problems in contrast to only partial or mild deviations in cognitive performance (Öhman et al., 2007; Österberg et al., 2009; Eskildsen et al., 2015; Pihlaja et al., 2022) or lack of any evidence for cognitive impairment (Österberg et al., 2009; McInerney et al., 2012; Deligkaris et al., 2014). Sometimes, unimpaired or mildly impaired cognitive performance is linked with a greater subjective cost associated with cognitive test performance in subjects with burnout than those without (Oosterholt et al., 2014; Wu et al., 2022). Previously, Diestel et al. found that burnout subjects performed at the same level as healthy controls when the test was easy, but in the more cognitively demanding test, their performance was significantly worse, which might explain the variance of the previous results (Diestel et al., 2013). On the other hand, there are studies reporting objective evidence for cognitive deficits in several cognitive functions in burnout (Sandström et al., 2005; Van Der Linden et al., 2005; van Dam et al., 2011; Oosterholt et al., 2012; Österberg et al., 2012; Jonsdottir et al., 2013; Eskildsen et al., 2015; Krabbe et al., 2017; Van Dijk et al., 2020; Lemonaki et al., 2021) and other studies showing structural changes in the prefrontal cortex and limbic brain structures (Blix et al., 2013; Savic, 2015; Chow et al., 2018), which are crucial for executive functions. Furthermore, some studies suggest that decreased brain-derived neurotrophic factor (BDNF) might explain the cognitive impairment in burnout (He et al., 2017; Chow et al., 2018). Despite frequent subjective reports of cognitive challenges and some objective evidence for cognitive deficits in burnout, objective evidence for brain dysfunction or alterations in brain physiology is scarce, and the number of electrophysiological studies is limited.

ERP studies have indicated alterations in both voluntary and involuntary attention in subjects with burnout (Luijtelaar et al., 2010; Sokka et al., 2014, 2016; Wu et al., 2022). These studies typically apply the classic or modified auditory oddball paradigm and assess the frontal P3a evoked by unexpected or novel stimuli and the parietal P3b evoked by target stimuli (Polich, 2007). Some electrophysiological evidence for alterations in attentional processing in burnout has been obtained, for example, reduced P3a amplitude to distractors suggesting impaired involuntary orienting to task irrelevant but potentially otherwise significant stimuli (Sokka et al., 2016) and reduced P3b amplitude to targets possibly reflecting diminished voluntary allocation of attentional resources to task-relevant stimuli (Luijtelaar et al., 2010; Sokka et al., 2017). In addition to alterations in attentional processes, alterations in error processing and negative feedback processing have been detected (Gajewski et al., 2017). Furthermore, dysfunctional fronto-parietal cognitive control in burnout has been suggested based on a reduction in working memory-related P3b over the posterior scalp with a simultaneous increase in amplitude over the frontal scalp (Sokka et al., 2016). The lateral prefrontal cortex is involved in voluntary attention and contributes to P3b when demanding cognitive operations are needed (Hartikainen and Knight, 2003). To that end, the topographic shift from more posterior to more anterior scalp sites in P3b may indicate an increased need for frontal cognitive control in a working memory task as a compensatory mechanism in subjects with burnout to maintain an adequate level of task performance. A similar anterior shift has been detected in older adults and has been suggested to reflect reliance on frontal compensatory mechanisms (Alperin et al., 2014).

While some of the previous ERP findings in subjects with burnout have been suggested to reflect alterations in frontal cognitive control, there is a lack of studies, where EFs frequently needed in everyday tasks, such as inhibitory control, task switching, and working memory, have been investigated in combination with brain physiology in subjects with burnout. Gajewski et al. (2017) studied EF and the underlying neural processes in subjects with preclinical burnout and found diminished frontal P3a in trials requiring working memory and task switching, indicating impairment of executive control. They also discovered that the amplitude of the parietally distributed P3b was generally reduced in subjects with subclinical burnout, even if there was no impairment in cognitive performance. Diminished P3b was interpreted as reduced cognitive resources allocated to the task, either due to neural dysfunction or a lack of motivation in subclinical burnout. A similar reduction in P3b, associated with impaired task performance, was observed by Sokka et al. (2017) in a task requiring shifting between attentional sets in subjects with severe burnout. The P3b reduction was speculated to be linked either with disrupted working memory processes or ineffective selection of what is relevant or irrelevant for the task. They suggested that severe burnout is associated with inadequate processing when rapid shifting of attention between tasks is required.

To this date, knowledge on the neural basis of burnout is lacking. With everyday tasks engaging multiple EF concurrently and subjects with burnout experiencing challenges in EF in daily life (Österberg et al., 2009; Oosterholt et al., 2012; Pihlaja et al., 2022), it is important to study brain functions during tasks requiring integration of different EFs. The objective of this study was to gain a better understanding of the effects of burnout on cognitive functions and brain physiology, specifically on EF and the neural processes underlying them. This study is part of a larger non-invasive neuromodulation study in the Sustainable Brain Health project assessing the impact of non-invasive transcutaneous vagus nerve stimulation (tVNS) on EF in healthy subjects and subjects with burnout. The results of tVNS on EF and the underlying neural processes will be presented in a separate publication. In this study, we focus only on the impact of burnout on brain physiology and EF.

We expected burnout to be associated with altered neural processes underlying EF and reflected in ERPs during a task engaging EF. We hypothesized that the neural processes underlying EF are inefficient, slowed down, or both. In the case of intact performance, increased P3 amplitude was expected, as greater P3 amplitude has been linked with increased effort and resource allocation. Greater N2 or P3 amplitudes during intact task performance would reflect the compensatory allocation of cognitive control or attentional resources to maintain the performance level. In case of impaired performance, the opposite pattern, i.e., diminished N2 and P3 ERP amplitudes, was expected. Slowed down or prolonged neural processes in EF task, on the other hand, would be detected as increased ERP peak (N2, P3) or peak-to-peak (N2-P3) latencies. From these ERP indices, derived during a task engaging several EFs, we also aimed to create a novel brain physiology-based biomarker of burnout.

## 2. Methods

### 2.1. Subjects

**Figure 1:**
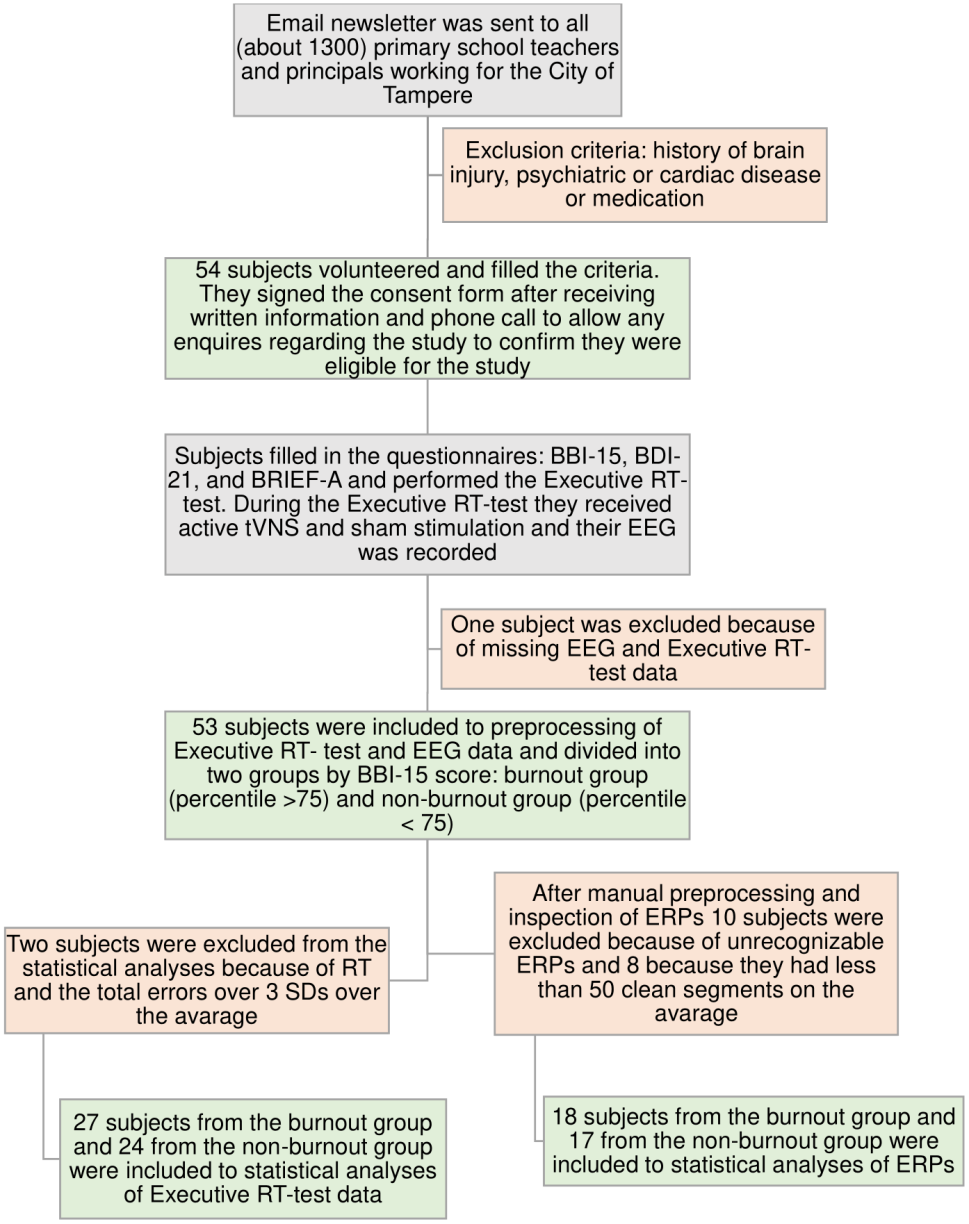
Flowchart of the subjects´ recruitment and intake processes. In the burnout group, 12 subjects had severe burnout, nine had moderate burnout, and five had mild burnout according to BBI-15 scores; in the non-burnout control group, there were only subjects without burnout.

Fifty-four teaching professionals were recruited for the study from the City of Tampere. This study was conducted in accordance with the Declaration of Helsinki and approved by the Ethics Committee of Tampere University Hospital (approval number: R20094). Participants provided their written informed consent to participate. The recruitment and intake process flowchart is presented in Figure 1.

### 2.2. Questionnaires

The Bergen Burnout Indicator 15 (BBI-15) consists of 15 claims related to three main symptoms of burnout: cynicism, lack of professional self-esteem, and exhaustion (Näätänen et al., 2003). The Beck Depression Inventory 21 (BDI-21) consists of 21 multiple-choice statements related to the symptoms of depression (Beck et al., 1996). The BRIEF-A is a clinically validated self-assessment tool consisting of 75 claims concerning EF in daily life. Responses are aggregated into nine clinical EF scales, two indices [metacognition (MI) and behavior regulation indices (BRIs)], and a global executive composite (GEC) derived by combining individual scales. The MI consists of initiation, working memory, planning or organization, task monitoring, and organization of materials scales, and the BRI consists of emotional control, shifting, inhibition, and self-monitoring scales. BRIEF-A raw scores are transformed into age-normalized T-scores based on a normative sample where T-score 50 is the mean value. The T-scores are used in analyses. T-scores over 65 points are considered abnormal, i.e., they differ by more than 1.5 standard deviations from the normative sample mean (Roth et al., 2005).

### 2.3. The Executive reaction time test

**Figure 2:**
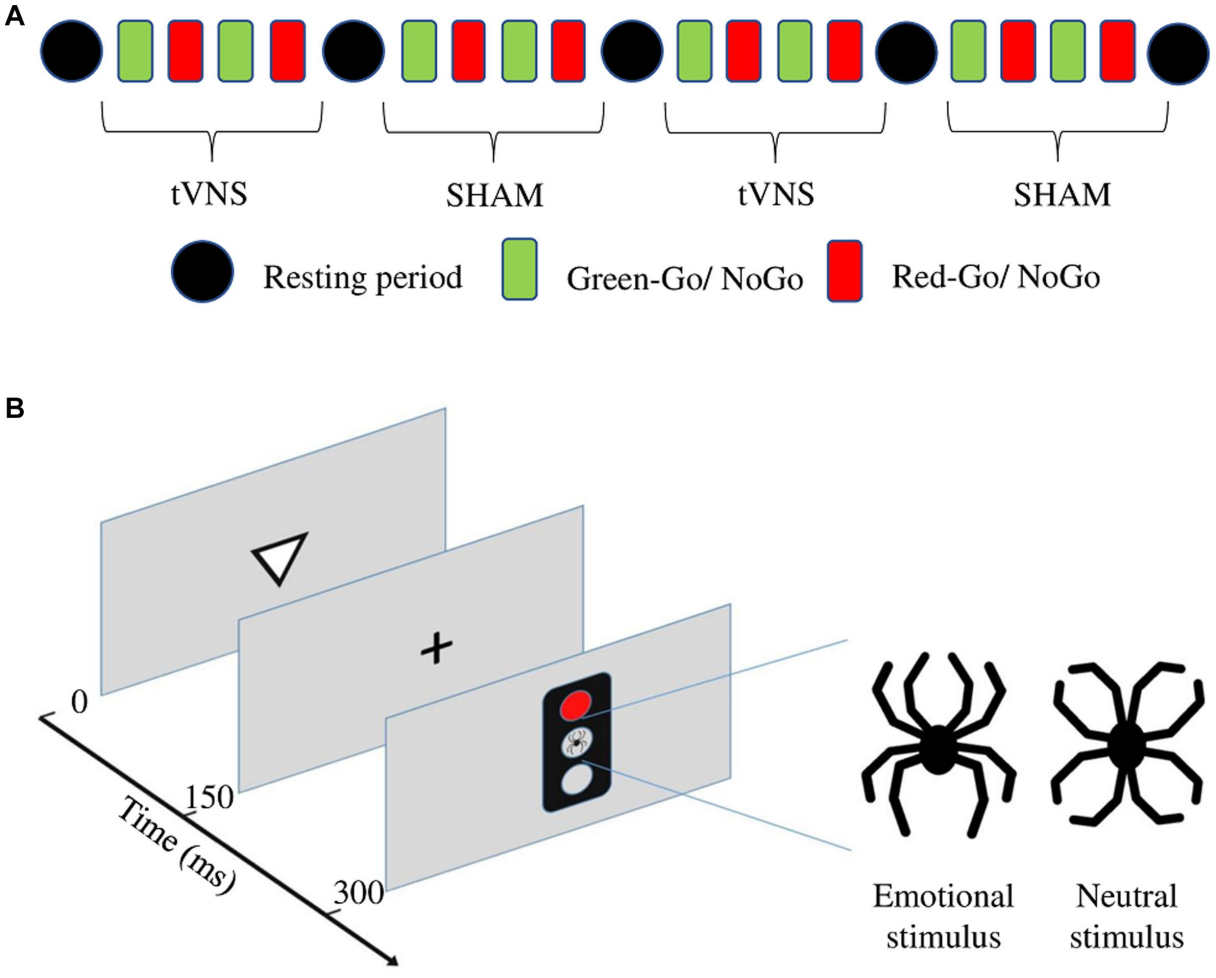
**(A)** Schematic presentation of the stimulation protocol during the Executive RT test. **(B)** Schematic presentation for one trial of the Executive RT test. The trial begins with a triangle presented in the middle of the screen for 150 ms and pointing up or down. A fixation cross appears for another 150 ms. Subsequently, a traffic light is presented for 150 ms with either a red or green light and either a neutral or emotional distractor in the middle. The distractor, a black line drawing resembling a spider (emotional distractor) or a flower (neutral distractor) is composed of identical line elements but in a different configuration. The subject is supposed to respond to the orientation of the previously presented triangle during a Go-trial and press a button with the middle finger if the triangle is pointing up and the index finger if it is pointing down. During a NoGo trial, the subject is supposed to refrain from responding.

A computer-based, Go-NoGo reaction time test, the Executive reaction time test (Executive RT test; Hartikainen et al., 2010), was used as an objective measure of executive functions. It is an integrated test of executive functions assessing controlled attention, working memory, set-shifting, and inhibition of prepotent responses and emotional distraction. The task engages multiple executive functions concurrently in the context of task-irrelevant, threat-related emotional stimuli, and tapping into diverse frontal circuits (Hartikainen et al., 2010). A schematic presentation of the stimulation protocol and the Executive RT test is presented in Figure 2.

Rather than aiming to isolate specific cognitive processes, the Executive RT test has been designed to be sensitive for detecting alterations in brain health in general, especially changes in EF in clinical populations. The task mimics everyday challenges for EF where multiple EFs are simultaneously engaged and integrated. Reaction times, different error types, and the impact of a threatening stimulus on them can be analyzed, providing versatile information about executive functions in general and about specific functions such as inhibition, working memory, and emotional control. The test has been shown to be relatively robust to the learning effect (Erkkilä et al., 2018) and sensitive to both improvement (Liimatainen et al., 2016; Sun et al., 2017) and impairment of EFs (Hartikainen et al., 2010, 2014).

The Executive RT test consists of four practice blocks and four test blocks, with a 4-min resting period between each block. Practice blocks are excluded from the analyses to remove the learning effect (Erkkilä et al., 2018). One block lasts approximately 2 min, and the complete test includes 36 min of active test time and 16 min of resting time. At the beginning of the experiment, NoGo trials and half of both were in the context of an emotional and half in the context of a neutral distractor. Subjects were told to react as fast and accurately as possible according to the response rule given before each block. The response rule determines which color of the traffic light, green or red, is the Go signal and which is the NoGo signal. The rule for responding changed after each block, i.e., if the green color was the Go signal and the red color the NoGo-signal in the first block, in the second block the rule was reversed, and the red was the Go signal and the green was the NoGo signal. The block consisted of 64 trials, where 32 trials were Go-trials and 32 trials were NoGo trials, and half of both were in the context of an emotional and half in the context of a neutral distractor (see Figure 2). One trial consisted of a triangle pointing up or down, a traffic light presenting either a red or green light and either a neutral or emotional distractor in the middle (see Figure 2B). The subject had to respond to the orientation of the presented triangle during a Go-trial by pressing an “up” button with the middle finger if the triangle was pointing up and by pressing the “down” button with the index finger if the triangle was pointing down. During a NoGo trial subject had to withhold responding. The distractor was a black line drawing resembling a spider (emotional distractor) or a flower (neutral distractor), and it was composed of identical line elements in a different configuration to control for visual differences. The distractor was presented in the middle of the central traffic light (Figure 2).

### 2.4. Electroencephalography and data processing

EEG was recorded using 64 Ag/AgCl active electrodes (actiCAP, Gilching, Germany), a QuickAmp amplifier, and Brain Vision Recorder software (Brain Products, GmbH) with a 500 Hz sampling rate. The impedance of all electrodes was kept below 5 kΩ throughout the recordings.

Brain Vision Analyzer software (version 2.1, Brain Products GmbH) was used for the preprocessing of EEG. EEG was re-referenced to linked mastoids, and the signal was band-pass filtered at 0.1–40 Hz. Blinks and other artifacts were removed using independent component analysis (ICA)-based correction. After ICA correction, signals with over 80 μV peak-to-peak voltage differences were removed from the analysis. EEG was epoched into 2,000-ms segments, starting 200 ms before and 1,800 ms after trial onset.

Finally, EEG segments were averaged for each condition (Go or NoGo), stimulator type (active or sham), and distractor type (emotional or neutral), resulting in eight different ERP conditions for each subject (in both Go and NoGo conditions: neutral active, neutral sham, emotional active, and emotional sham). Based on convention (Luck, 2005; Boudewyn et al., 2018), previous experience with this paradigm, and visual inspection of the individual ERP averages, a minimum of 50 artifact-free EEG epochs were required for each ERP condition for the subject to be included in the analysis. The number of trials in different conditions was comparable within each subject included in the analysis.

The N2 component after the Go/NoGo signal was identified from the averaged waveforms using semiautomatic peak detection and visual inspection. N2 was defined as the most negative peak in the timeframe from 200 ms to 350 ms and P3 as a subsequent positive peak appearing between 300 and 500 ms after the traffic light, i.e., 500–650 ms and 600–800 ms from the trial onset, respectively. Amplitudes were counted from the averaged baseline of 200 ms before trial onset. Due to the frontal distribution of the N2 peak (Folstein and Van Petten, 2008), we used frontal channels Fz, F1, F2, F3, and F4 to measure N2 peak amplitude and CPz, CP1, CP3, CP2, and CP4 to measure P3 amplitude, reflecting task-related attentional resources (Polich, 2007). We focused on the frontal N2 amplitude during NoGo condition, thought to reflect cognitive control required in response inhibition (Folstein and Van Petten, 2008), and centroparietal P3 during Go conditions, thought to reflect attentional resource allocation required in target detection (Kok, 2001; Polich, 2007).

### 2.5. Statistical analyses

For statistical analyses, average N2 amplitude and latency were calculated for channels F1, F2, F3, F4, and Fz, and for P3 amplitude and latency for channels CP1, CP2, CP3, CP4, and CPz. The N2-P3 IPL was calculated by subtracting N2 latency from P3 latency on each channel separately and then averaged similarly to other amplitudes and latencies over frontal and CP channels. Data were checked for normality with the Shapiro–Wilk normality test. As the distributions of reaction times, amplitudes, and latencies were skewed, a Kruskal–Wallis rank-sum test was used for statistical analyses. “Group” (burnout vs. non-burnout) was used as a factor in reaction time, error, and ERP analyses. Because the effect of stimulator status was not assessed in this article, “ON” and “OFF” conditions were averaged for the analyses. ERP analysis was conducted separately for Go and NoGo conditions.

Errors were analyzed with a generalized mixed-effects logistic regression (Dixon, 2008; Jaeger, 2008). Each error type was analyzed using a separate model predicting the probability of making that kind of error. “Subject” was used as a random effect predictor, and “group,” “stimulator status,” and “emotional valence” were used as fixed effect predictors. The logistic regression trial outcomes were dichotomized into binary classes so that for total errors, classes were “correct” (correct button press in Go-trial or no response in NoGo trial) or “error” (incorrect or missing button press in Go-trial or any button press in NoGo trial); for incorrect responses, “incorrect” (incorrect button press) or “other” (correct or missing button press); for missed responses, “miss” (missing button press) or “other” (correct or incorrect button press); and for commission errors, “commission error” (a button was pressed in NoGo trial) or “correct” (no button press in NoGo trial).

Correlation analyses were done using Spearman’s rank correlation. To assess how many electrophysiological parameters (P3 amplitude and latency and N2-P3 IPL) explained the variance of the questionnaire scores, a linear regression model with previous parameters as predictors was created.

Statistical analysis was done using R statistics v. 3.1.1 (R Core Team, 2022). Repeated measures ANOVA was done with EZ package v. 4.2–2 (Lawrence, 2016) and regression analysis with lme4 package v. 1.1–10 (Bates et al., 2015).

## 3. Results

### 3.1. Inventories

Groups differed significantly in all BRIEF-A indices, BDI-21, and BBI-15 (Table 1).

**Table 1:** Age, education, BBI score, BDI score, and BRIEF-A indexes between groups included in the statistical analyses of the Executive RT test. BDI score reflected mild depression in burnout groups and no-depression in non-burnout groups. Based on the BBI-15 scores, 26 women and 2 men were assigned to the burnout group, and 23 women and 2 men were assigned to the non-burnout group.

	Group	Mean (SD)	*p*
Age (years)	Burnout	44.56 (9.39)	0.84
	Non-burnout	44.00 (10.36)	
Education (years)	Burnout	18.96 (2.29)	0.28
	Non-burnout	18.26 (2.18)	
BBI score	Burnout	60.04 (11.29)	<0.00
	Non-burnout	33.17 (7.50)	
BDI score	Burnout	15.93 (7.69)	<0.00
	Non-burnout	4.96 (3.76)	
BRI	Burnout	58.85 (10.91)	<0.00
	Non-burnout	46.25 (9.86)	
MI	Burnout	61.37 (10.47)	<0.00
	Non-burnout	48.46 (10.29)	
GEC	Burnout	60.70 (10.25)	<0.00
	Non-burnout	47.21 (9.56)	

### 3.2. Executive reaction time test

There were no significant differences between burnout and non-burnout group**s** in reaction times (burnout = 358 ms (81 ms), non-burnout = 401 ms (107 ms), ꭓ^2^ = 2.22, *p* = 0.14), or in any error type**s** (Tables 2, 3).

**Table 2:** Odds ratios and confidence intervals for total errors, incorrect responses, commission errors, and missing responses between groups.

Variable	OR	95% CI
Total errors	1.01	0.62–1.64
Incorrect responses	0.98	0.50–1.92
Commission errors	0.83	0.44–1.55
Missing responses	0.93	0.31–2.82

**Table 3:** Medians and IQRs for errors.

Executive RT test	Group (n)	Median (IQR)
Total errors (%)	Burnout (27)	1.56 (1.89)
	Non-burnout (24)	1.32 (0.78)
Incorrect responses (%)	Burnout (27)	0.98 (1.38)
	Non-burnout (24)	0.78 (1.56)
Missing responses (%)	Burnout (27)	0.21 (0.61)
	Non-burnout (24)	0.20 (0.78)
Commission errors (%)	Burnout (27)	1.17 (1.73)
	Non-burnout (24)	0.78 (1.22)

### 3.3. Event-related potentials

A total of 16 women and 2 men in the burnout group and 15 women and 2 men in the non-burnout group met the quality and quantity criteria for ERP analysis. In the burnout group, eight subjects had severe, six had moderate, and four had mild burnout according to BBI-15 scores, and in the non-burnout control group, there were only subjects without burnout. Age, educational level, and questionnaire scores of the groups included in the ERP analyses are presented in Table 4. A separate behavioral analysis was done for the ERP subgroup. The results were in line with whole-group analyses, with no statistically significant differences found in reaction times or error rates.

**Table 4:** Age, education, BBI scores, BDI scores, and BRIEF-A indexes for groups included in statistical analyses of ERPs. Sixteen women and 2 men in the burnout group and 15 women and 2 men in the non-burnout group met the quality and quantity criteria for ERP analysis.

	Group	Mean (SD)	*p*
Age (years)	Burnout	46.44 (8.96)	0.83
	Non-burnout	45.76 (9.71)	
Education (years)	Burnout	19.17 (2.01)	0.56
	Non-burnout	18.75 (2.11)	
BBI score	Burnout	60.06 (10.34)	< 0.001
	Non-burnout	33.06 (8.47)	
BDI score	Burnout	15.89 (7.12)	< 0.001
	Non-burnout	4.12 (2.93)	
BRI	Burnout	58.94 (11.58)	0.00
	Non-burnout	46.18 (9.86)	
MI	Burnout	63.17 (9.98)	< 0.001
	Non-burnout	47.47 (9.86)	
GEC	Burnout	62.06 (10.08)	< 0.001
	Non-burnout	46.53 (9.84)	

#### 3.3.1. ERP amplitudes—frontal N2 and centroparietal P3

**Figure 3:**
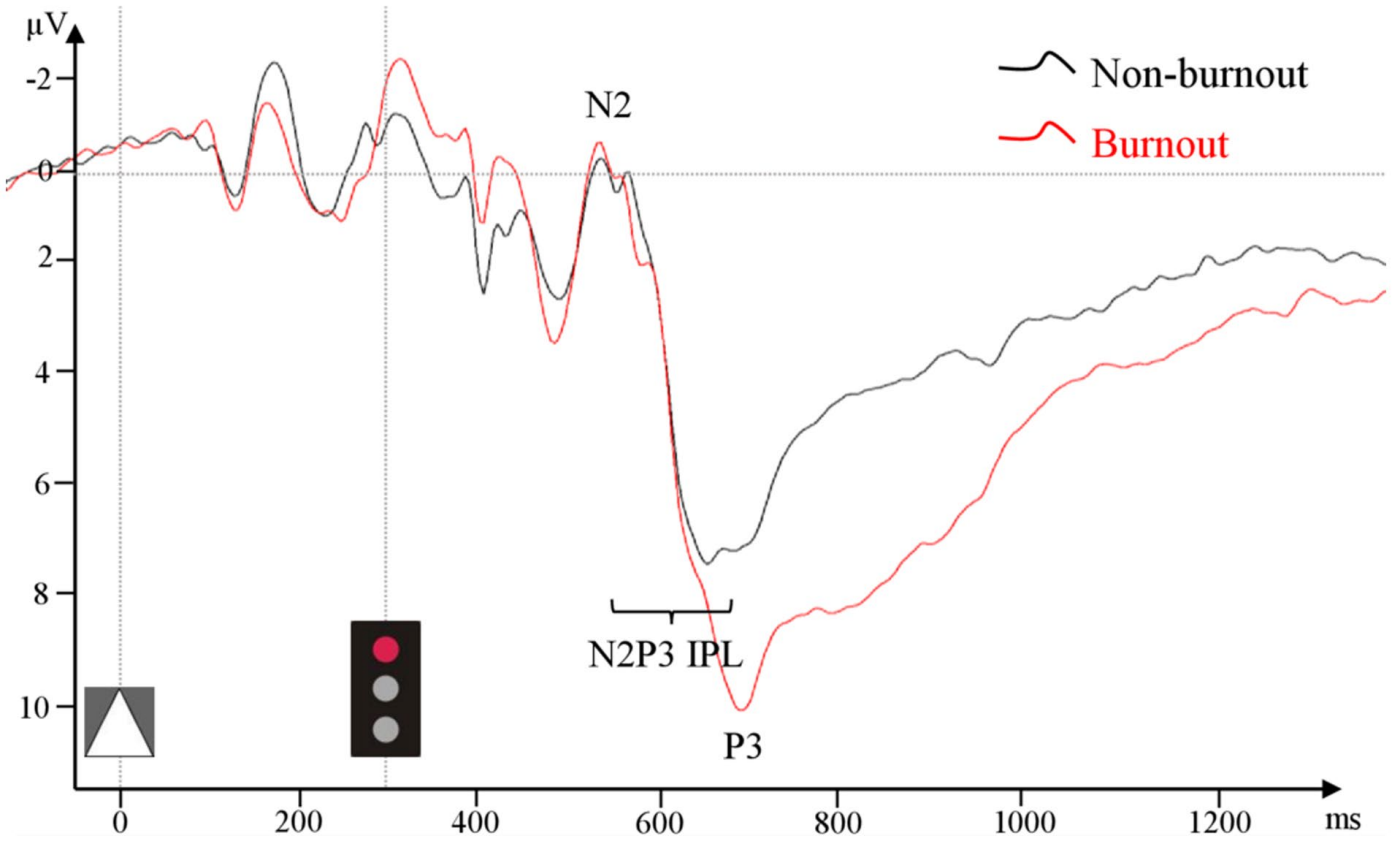
ERP grand average from pooled CP1–CP4 and CPz in the Go condition for the burnout and non-burnout groups separately.

There were no statistically significant differences between groups in frontal N2 amplitude in the Go or NoGo condition. Centroparietal (CP) P3 amplitude in the Go condition was significantly larger in the burnout group (9.89 μV (5.69 μV)) compared to the non-burnout group (6.73 μV (3.48 μV), ꭓ^2^ = 5.49, *p* = 0.02, Figure 3). In the NoGo condition, centroparietal (CP) P3 did not differ between groups (burnout = 6.44 μV (5.57 μV), non-burnout = 6.14 μV (3.58 μV), ꭓ^2^ = 0.68, *p* = 0.41).

#### 3.3.2. ERP latencies—N2 and P3 peak and N2-P3 interpeak latencies

Centroparietal (CP) N2-P3 interpeak latency (CP N2-P3 IPL) in Go condition differed significantly between groups (burnout = 188.20 ms (85.40 ms), non-burnout = 135.00 ms (49.40 ms), ꭓ^2^ = 5.17, *p* = 0.02, Figure 3). There was no difference in frontal or centroparietal (CP) N2 and P3 single- or interpeak latencies.

### 3.4. Correlation and regression analysis

Spearman’s rank correlation analysis was done for the CP P3 amplitude, latency, and N2-P3 IPL in the Go condition and main questionnaire indices. Correlation analyses showed significant positive correlations between the P3 amplitude and the BBI score (ρ = 0.45, *p* = 0.01) and MI (ρ = 0.47, *p* = 0.01). Furthermore, there were significant correlations between the CP P3 latency in the Go condition and the MI index from BRIEF-A (ρ = 0.36, *p* = 0.03). All correlation coefficients and *p*-values are presented in Table 5.

**Table 5:** Spearman’s rank correlation coefficients and statistical significances for CP P3 amplitude, latency, and CP N2-P3 interpeak latency in go condition between BBI-, BDI-, and BRIEF-A results.

	CP P3 Go amplitude	CP P3 Go latency	CP N2-P3 Go IPL
BBI score	0.45 (0.01)	0.09 (0.61)	0.36 (0.05)
BDI score	0.32 (0.07)	0.06 (0.74)	0.36 (0.04)
GEC	0.33 (0.06)	0.29 (0.09)	0.45 (0.01)
MI	0.47 (0.01)	0.36 (0.03)	0.51 (0.00)
BRI	0.16 (0.39)	0.07 (0.70)	0.21 (0.25)

To assess how much physiological measures, CP P3 Go amplitude and latency, explained BBI score and BRIEF questionnaire score variances, linear regression models were created using CP P3 amplitude and latency in the Go condition as predictors (see Table 6). CP P3 amplitude alone predicted BBI score significantly: R^2^ for the BBI score was 21% (*p* = 0.01). Furthermore, using P3 amplitude and latency together as predictors, the multiple R^2^ for the BBI score was 25% (*p* = 0.01). In addition, P3 amplitude and latency significantly predicted MI of BRIEF-A: R^2^ was 36% (*p* < 0.00).

**Table 6:** Regression model summaries. Centroparietal (CP) P3 amplitude and latency were used to model the BBI-15 score and BRIEF-A metacognition index (MI). Panel (A) model summary and panel (B) coefficient summary.

(A) Model summary	BBI-15	MI
F	(2, 29) = 4.90	(2, 29) = 12.69
P	0.01	< 0.01
R^2^	0.25	0.47
Adj. R^2^	0.20	0.43

(B) Coefficient summary	BBI-15				MI			
	Est.	S.E.	t val.	*p*	Est.	S.E.	t val.	*p*
(Intercept)	−15.67	34.81	−0.45	0.66	−32.39	25.49	−1.27	0.21
CP P3 amplitude	1.71	0.68	2.50	0.02	1.27	0.53	2.38	0.02
CP P3 latency	0.07	0.05	1.29	0.21	0.11	0.04	2.96	0.01

In addition to single peak P3 amplitude and latency in the Go condition, a regression model was created for CP P3 amplitude and CP N2-P3 IPL in the Go condition (Table 7). The R^2^ for BBI score was 39% (*p* = 0.00), and for MI of BRIEF-A, R^2^ was 47% (*p* < 0.00).

**Table 7:** Regression model summaries. Centroparietal (CP) P3 amplitude and N2-P3 interpeak latency were used to model the BBI-15 score and BRIEF-A metacognition index (MI). Panel (A) model summary and panel (B) coefficient summary.

(A) Model summary	BBI-15	MI
F	(2, 27) = 8.49	(2, 29) = 12.69
P	0.001	< 0.001
R^2^	0.39	0.47
Adj. R^2^	0.34	0.43

(B) Coefficient summary	BBI-15				MI			
	Est.	S.E.	t val.	*p*	Est.	S.E.	t val.	*p*
(Intercept)	12.62	8.25	1.53	0.14	23.69	6.43	3.69	0.00
CP P3 amplitude	1.61	0.66	2.44	0.02	1.35	0.51	2.64	0.01
CP N2-P3 IPL	0.11	0.04	2.84	0.01	0.11	0.03	3.79	0.00

## 4. Discussion

The current study aimed to better understand the impact of burnout on executive functions and the underlying neural processes. Despite unimpaired cognitive performance, subjects with burnout experienced challenges in EF in daily life. Uncompromised cognitive performance was linked with significantly larger P3 amplitude and prolonged N2-P3 IPL (Go condition, CP region). Prolonged N2-P3 IPL may reflect slowed neural processes or prolonged transitions from one process to another in the later phase of the task requiring cognitive control. Increased P3, on the other hand, may reflect inefficient neural processes underlying EFs and the consequent need to allocate more neural processing resources to achieve the same cognitive performance level as healthy controls. We suggest that burnout is linked to inefficient and/or sluggish neural processing underlying EF.

We also discovered that P3 amplitude and N2-P3 interpeak latencies obtained during an EF task correlated significantly with the severity of burnout. Furthermore, a regression model using CP P3 amplitude and the N2-P3 IPL latency as predictors significantly predicted the BBI-15 score. In other words, brain physiology-derived indices predicted the level of burnout symptoms experienced. We suggest that the combination of P3 amplitude and N2-P3 IPL measured during a task engaging several EFs shows promise as an objective brain physiology-based biomarker for burnout.

The P3 amplitude, the P3 latency, and the CP N2-P3 IPL all correlated with the metacognition index of BRIEF-A, with more challenges in metacognition linked with greater P3 amplitude and longer P3 and N2-P3 interpeak latencies. EF and metacognition have a crucial role in successful functioning in daily life and the ability to work in a modern information society. On the other hand, subtle alterations in EF and metacognition often escape objective testing. To this end, it is of great interest that we found objective electrophysiological indices of subjective challenges in metacognition in daily life. Future studies need to confirm the reliability and generalizability of these findings to other groups, but if confirmed, they have huge implications for improved detection of challenges in higher cognitive control functions and the underlying neural dysfunction in burnout, independent of the etiology of cognitive dysfunction. The efficiency of metacognition is tightly linked with many factors that improve or impair brain health, including burnout. For example, we have shown previously that heart rate variability (HRV) during sleep, a measure reflecting the quality of sleep and how recovering the sleep is, correlates with MI (Pihlaja et al., 2022). With EF and brain health tightly linked, electrophysiological indices of metacognition would also be beneficial in intervention studies aiming at improved brain health.

According to the BRIEF-A questionnaire, subjects with burnout experienced more challenges in their daily tasks requiring EF, but in objective testing, their performance did not differ from the performance of subjects without burnout. This concords with the findings by Oosterholt et al. and Krabbe et al., who reported that subjects with and without burnout performed at the same level, but subjects with burnout reported higher subjective cost (exhaustion) compared to the healthy controls (Oosterholt et al., 2014; Krabbe et al., 2017). This indicates that subjects with burnout had to allocate more neural resources to achieve the same performance level, which could be linked to the increased P3 amplitude (Kok, 2001) observed in this study. We speculate that the increased P3 amplitude in this study reflects compensatory mechanisms due to inefficient neural processes linked with EF.

Previously, P3b reductions have been reported in studies assessing EF, specifically set sifting, in burnout (Gajewski et al., 2017; Sokka et al., 2017). Sokka et al. (2017) found the greatest reduction in P3b in subjects with severe burnout and compromised task performance. Gajewski et al. (2017), on the other hand, found diminished P3b despite equal performance in subclinical burnout. In the current study, a non-clinical sample of currently working subjects with burnout symptoms showed increased parietal P3, i.e., P3b, in the Go condition along with uncompromised task performance. As P3 amplitude is influenced by many different subject-, stimulus-, and task-related factors and several overlapping cognitive processes (Polich, 2007), direct comparisons between results from studies using different tasks are challenging. There are several methodological differences between the previous studies and the current study. The most obvious difference is the task, but there are also some differences in the subject populations and the assessment of burnout. Unlike in the previous studies, a set-shifting task was not used in the current study, but rather an integrated test of EF, where sets were shifted only a few times during the entire experiment (between blocks), in contrast to constant, rapid, and frequent set-shifting (between trials) in the studies of Sokka et al. (2017) and Gajewski et al. (2017).

There are several cognitive processes temporarily overlapping and reflected in the P3 peak amplitude, and the processes differ depending on the task. While the impact of burnout on P3 amplitudes was opposite in the previous studies and the current study, the results fit with the general framework of P3 amplitude reflecting the amount of resource allocation. Reduced P3 amplitude along with impaired performance in subjects with severe burnout may indicate inefficient neural processing related to EF in combination with inadequate allocation of resources to maintain intact task performance (Sokka et al., 2017). On the other hand, neural processing in subjects with subclinical burnout may be efficient enough to maintain intact performance even with less neural resources allocated to the task (Gajewski et al., 2017). Another possibility is that a lack of sensitivity in performance measures does not allow for subtle decrements in performance. Interestingly, the current results with increased P3 in burnout resemble results from an ERP study on compensatory mechanisms in normal cognitive aging, with high-performing healthy older adults showing increased P3 amplitude (Riis et al., 2008). We suggest increments or decrements in P3b amplitude reflecting increased or diminished allocation of attentional resources may be observed in burnout depending on the severity of burnout, task demands, motivational factors, and the efficiency of neural processes needed to perform the task. Inadequate allocation of resources, as indexed by diminished P3b, is typically linked with reduced performance levels. While compensatory mechanisms allowing for intact performance despite impaired or inefficient neural processing are linked with additional allocation of processing resources indexed by increased P3b.

To our knowledge, this is the first study to assess the effect of burnout on ERP interpeak latencies and, more specifically, to report N2-P3 IPL along with P3 amplitude to correlate with burnout symptoms. While the P3 latency did not significantly differ between the groups, the time between the centroparietal (CP) N2 and P3 peaks, CP N2-P3 IPL in the Go condition, was prolonged in the burnout group. This could reflect slowed cognitive processing speed due to burnout, which has been associated with burnout in previous studies (Öhman et al., 2007; Österberg et al., 2009; Jonsdottir et al., 2013; Eskildsen et al., 2015). The N2-P3 IPL isolates the speed of neural processes linked with cognitive functions that occur after the N2 peak and before the P3 peak, while the more traditionally used P3 latency reflects the speed of all the neural processes from early sensory processing to later cognitive processing occurring during the time elapsed from stimulus presentation to the P3 peak. Thus, N2-P3 IPL may be thought to reflect the speed of later cognitive processes, including transition time from cognitive processes linked with N2 to those linked with P3.

In the current study, N2-P3 IPL proved to be superior to P3 latency in reflecting burnout symptoms and as part of a burnout prediction model. Even though the P3 latency did not result in a statistically significant difference between the groups, it is feasible that prolonged P3 latency may have contributed to prolonged IPL. The P3 latency reflects the time elapsed for all cognitive processes preceding P3, and thus, there may be more variability in P3 than in IPL latency, where the time for preceding processes has been subtracted away. To that end, IPL latency with a smaller variance may require a smaller sample size to reach statistical significance than P3 latency with a higher variability. The N2-P3 IPL shows promise as an independent biomarker of burnout as well as in combination with other electrophysiological indices. With very few studies focusing on interpeak latencies, based on the current results, we recommend future electrophysiological studies to assess IPLs alongside the more traditional latency and amplitude measures.

The study’s limitations relate to assessing the effects of burnout on EF and ERPs while subjects received tVNS and placebo stimulation and to gender balance. Based on our preliminary analysis of the impact of tVNS on ERPs, which will be reported in a separate publication, the effects found in this study due to burnout cannot be attributed to the differential impact of tVNS on these groups. However, the effects of tVNS on results cannot be fully excluded, and in future, it is important to reproduce the study without tVNS. Furthermore, technical challenges in recording high-quality EEG during active electrical stimulation reduced our sample size. In addition, even though the sex balance was equal between the groups, it was unbalanced within the groups, with only two men in both groups. There may be differences in brain physiology between sexes, so in future studies, the sex balance should be better addressed.

The main strength of the current study relates to the simultaneous measurement of ERPs and cognitive performance in a task engaging several EFs. ERPs reflect rapid mental functions and the underlying brain physiology, as well as their alterations, in burnout. ERPs enable the detection of possible changes in the neural processing underlying EF. Even if subjects’ performance is not altered, changes in the underlying physiological process, such as increased P3 amplitude or latencies, can be observed. Performance measures, such as RT and errors, are coarse and temporally more limited than ERP, with temporal resolution in the millisecond range. Moreover, ERPs and interpeak latencies allow the assessment of the speed of specific cognitive processes or transitions from one cognitive process to another in contrast to a reaction time, which reflects the time elapsed for all sensory, cognitive, and motor processes required to react to a stimulus. In addition, ERPs provide information even when there are no behavioral counterparts. For example, in a successful NoGo situation, ERP reflects the neural processes needed to withhold a response even though there is no behavioral action.

In summary, we discovered that neural processing and related brain physiology underlying executive functions are altered in burnout, providing a better understanding of the challenges experienced in higher cognitive functions. We found that P3 amplitude at the CP region during the EF task reflects burnout symptoms as well as challenges in metacognition. The current study introduces N2-P3 IPL as an electrophysiological indicator of executive functions and their alterations due to a brain disorder, such as burnout. Furthermore, we created a regression model with P3 and N2-P3 IPL as predictors that link the BBI-15 burnout score to brain physiology. Finally, we showed evidence for burnout to be a distinct neuropsychiatric brain disorder with alterations in brain physiology and neural processes underlying cognitive functions.

## 5. Conclusion

Subjects with burnout had challenges with EF in daily life and altered cognitive control-related neural processing. Burnout decelerated transitions between cognitive processes underlying EF. To our knowledge, this is the first study using N2-P3 IPL in burnout subjects. The N2-P3 IPL measured during a task requiring cognitive control could be a generic and sensitive indicator of the efficiency of higher cognitive control functions in general and allow for intervention studies aiming to optimize brain health and EF.

Electrophysiological measures, P3 and IPL, could be used as predictors in the regression model to predict the severity of burnout and the efficiency of metacognition. To that end, we suggest burnout to be a neuropsychiatric brain disorder with alterations in cognitive control functions and corresponding brain physiology. Furthermore, we present novel brain physiology-based biomarkers of burnout.

## Data availability statement

The datasets presented in this article are not readily available because of hospital privacy policy and laws, which prohibit sharing the datasets outside European Union (EU) and European Economic Area (EEA) countries. Requests to access the datasets should be directed to KH, kaisa.hartikainen@live.com.

## Ethics statement

The studies involving humans were approved by Ethics Committee of Tampere University Hospital. The studies were conducted in accordance with the local legislation and institutional requirements. The participants provided their written informed consent to participate in this study.

## Author contributions

MP was responsible for the recruitment of subjects, data collection, statistical analysis and writing the manuscript. JP was responsible for creating the regression model, contributed to statistical analysis in general and participated in writing. E-HE, ET, and MV contributed equally on the data collection and participated in writing. Principal Investigator, KH was responsible for the experimental design and supervision of the data collection, analysis, interpretation of results, and writing. All authors contributed to the article and approved the submitted version.

## Funding

This research was funded by the Finnish Ministry of Social Affairs and Health from the European Social Fund’s (ESF) Program for Sustainable Growth and Jobs 2014–2020 Finnish structural fund (Sustainable Brain Health, S21966) and partly supported by the State funding for university-level health research, Tampere University Hospital, Wellbeing services county of Pirkanmaa.

## Conflict of interest

The authors declare that the research was conducted in the absence of any commercial or financial relationships that could be construed as a potential conflict of interest.

## Publisher’s note

All claims expressed in this article are solely those of the authors and do not necessarily represent those of their affiliated organizations, or those of the publisher, the editors and the reviewers. Any product that may be evaluated in this article, or claim that may be made by its manufacturer, is not guaranteed or endorsed by the publisher.
